# The effects of proprioceptive weighting changes on posture control in patients with chronic low back pain: a cross-sectional study

**DOI:** 10.3389/fneur.2023.1144900

**Published:** 2023-05-19

**Authors:** Xue Cheng, Jiajia Yang, Zengming Hao, Yan Li, Ruochen Fu, Yao Zu, Jinjin Ma, Wai Leung Ambrose Lo, Qiuhua Yu, Guifang Zhang, Chuhuai Wang

**Affiliations:** ^1^Department of Rehabilitation Medicine, The First Affiliated Hospital, Sun Yat-sen University, Guangzhou, China; ^2^Guangdong Engineering and Technology Research Center for Rehabilitation Medicine and Translation, The First Affiliated Hospital, Sun Yat-sen University, Guangzhou, China

**Keywords:** chronic low back pain, posture control, proprioception, proprioceptive weighting, center of pressure

## Abstract

**Introduction:**

Patients with chronic low back pain (CLBP) exhibit changes in proprioceptive weighting and impaired postural control. This study aimed to investigate proprioceptive weighting changes in patients with CLBP and their influence on posture control.

**Methods:**

Sixteen patients with CLBP and 16 healthy controls were recruited. All participants completed the joint reposition test sense (JRS) and threshold to detect passive motion test (TTDPM). The absolute errors (AE) of the reposition and perception angles were recorded. Proprioceptive postural control was tested by applying vibrations to the triceps surae or lumbar paravertebral muscles while standing on a stable or unstable force plate. Sway length and sway velocity along the anteroposterior (AP) and mediolateral (ML) directions were assessed. Relative proprioceptive weighting (RPW) was used to evaluate the proprioception reweighting ability. Higher values indicated increased reliance on calf proprioception.

**Results:**

There was no significant difference in age, gender, and BMI between subjects with and without CLBP. The AE and motion perception angle in the CLBP group were significantly higher than those in the control group (JRS of 15°: 2.50 (2.50) vs. 1.50 (1.42), JRS of 35°: 3.83 (3.75) vs. 1.67 (2.00), *p*_JRS_ < 0.01; 1.92 (1.18) vs. 0.68 (0.52), *p*_TTDPM_ < 0.001). The CLBP group demonstrated a significantly higher RPW value than the healthy controls on an unstable surface (0.58 ± 0.21 vs. 0.41 ± 0.26, *p* < 0.05). Under the condition of triceps surae vibration, the sway length (*p*_stable_ < 0.05; *p*_unstable_ < 0.001), AP velocity (*p*_stable_ < 0.01; *p*_unstable_ < 0.001) and ML velocity (*p*_unstable_ < 0.05) had significant group main effects. Moreover, when the triceps surae vibrated under the unstable surface, the differences during vibration and post vibration in sway length and AP velocity between the groups were significantly higher in the CLBP group than in the healthy group (*p* < 0.05). However, under the condition of lumbar paravertebral muscle vibration, no significant group main effect was observed.

**Conclusion:**

The patients with CLBP exhibited impaired dynamic postural control in response to disturbances, potentially linked to changes in proprioceptive weighting.

## 1. Introduction

Low back pain (LBP) is a major cause of disability in both developed and developing countries. It is among the top seven most serious health issues contributing to disability-adjusted life years (1, 2). Most patients with LBP are non-specific without specific pathological changes (3).

Sensorimotor impairment is considered an important contributor to LBP (4). Sensorimotor control refers to the complex process through which the central nervous system integrates and processes sensory input information derived from visual, vestibular, and somatosensory sources, and subsequently produces motor output. Numerous studies have confirmed the presence of motor control deficits in patients with chronic low back pain (CLBP), such as prolonged muscle reflex latencies and altered muscle recruitment patterns, which manifest as poor postural and motor control (5).

Sensory input is an important factor that influences postural and motor controls. The central nervous system assigns weights dynamically to sensory information from each peripheral input system. In the absence of sensory information, or when less accurate sensory information is detected due to disease or trauma, the central nervous system increases the weight of information from other body parts to achieve balanced control (6). Proprioceptive degeneration of the lumbar spine in patients with CLBP has been reported previously (7). As to whether such changes may contribute to inter- or intra-sensory information weight shifts, it has been suggested that peripheral sensory input does not increase vestibular or visual weight during postural control (8). This indicates that sensory information reweighting in patients with CLBP occurs mainly within the somatosensory system. The somatosensory system mainly includes proprioception and tactile sensation, of which proprioception is considered the most critical source of somatosensory information for posture and motion control. Proprioceptive information is important for neuromuscular control, which is essential for limb stability, postural control, and the discrimination of absolute and relative positions of the body (9). For example, patients with large-fiber sensory neuropathies lose proprioceptive inputs from the neck downward, while their motor nerves remain intact (10). They feel that they are floating in the air and are unable to move (10). Moreover, reweighting proprioceptive signals adaptively in demanding situations and conflicting are critical for posture control (11). For example, ankle proprioception is reliable on a stable surface, whereas weighting is shifted to the lumbar region on an unstable surface. It has been reported that gymnasts weigh sensory cues faster than healthy people (12). However, little is known about the ability to alter postural strategies without increasing the risk of stability in patients with CLBP. It has been proposed that patients with CLBP exhibit significant center of pressure (CoP) sway during sensory perturbation (11, 13). Non-linear measurements demonstrated that aging fallers exhibit more CoP motion after removing sensory perturbation (14). However, it is unclear whether patients with CLBP take longer to adapt to changing sensory conditions and dynamic perturbations in balance control.

Given the above, it remains unclear how patients with CLBP reweigh proprioception adaptively to changing postural conditions and respond to perturbation. This study aimed to investigate proprioceptive weighting changes and their influence on dynamic posture control in patients with CLBP.

## 2. Materials and method

### 2.1. Sample population

The participants were recruited through advertisements and were aged between 18 and 50 years, with or without CLBP. The inclusion criteria for patients with LBP were as follows: (1) medical diagnosis of non-specific LBP with pain and symptoms persisting for more than 3 months in the last 12 months and (2) LBP score >2 according to the visual analog scale (VAS). The inclusion criteria for healthy controls were: (1) no history of LBP in the last 12 months and (2) no pain for other reasons at the time of the visit. The exclusion criteria for all the participants were as follows: (1) prior spinal surgery, (2) spinal fractures or tumors, (3) LBP with neuropathy or radiculopathy, (4) history of neurological or musculoskeletal impairment, and (5) pregnancy. The participants were requested to attend a single testing session of 45–60 min duration to complete the questionnaires and proprioceptive assessments. This study was approved by the Human Subjects Ethics Subcommittee of the first affiliated hospital of Sun Yat-sen University (Grant No. 2021886). The study was also enrolled in the Chinese Clinical trial, registration number ChiCTR2200064270. All the patients recruited in the study were provided for written informed consent before participating in the study. The investigation was conducted according to the principles expressed in the Declaration of Helsinki.

### 2.2. Procedure

After providing written informed consent, the participants completed questionnaires that included questions on their demographic characteristics and history of LBP. Then, they underwent lumbar proprioception and proprioceptive postural control tests.

#### 2.2.1. Questionnaires

The visual analog scale (VAS) was adopted to evaluate LBP. It consists of a 100 mm horizontal line, with the right side signifying “worst imaginable pain” and the left side signifying “no pain” (15). The participants were asked to make a mark on the line that best represents their current level of pain, with higher numbers indicating more intense pain (16).

The Chinese version of the Roland-Morris Disability Questionnaire (RMDQ) was used to classify disabilities associated with LBP (17). The questionnaire measured the effects of LBP on daily activities, with scores ranging from 0 (no impairment) to 24 (severe impairment). Disability was categorized into three levels of severity based on the total score: mild (0–8), moderate (9–16), and high (17–24) (17).

#### 2.2.2. Lumbar proprioception measurement

**Figure 1:**
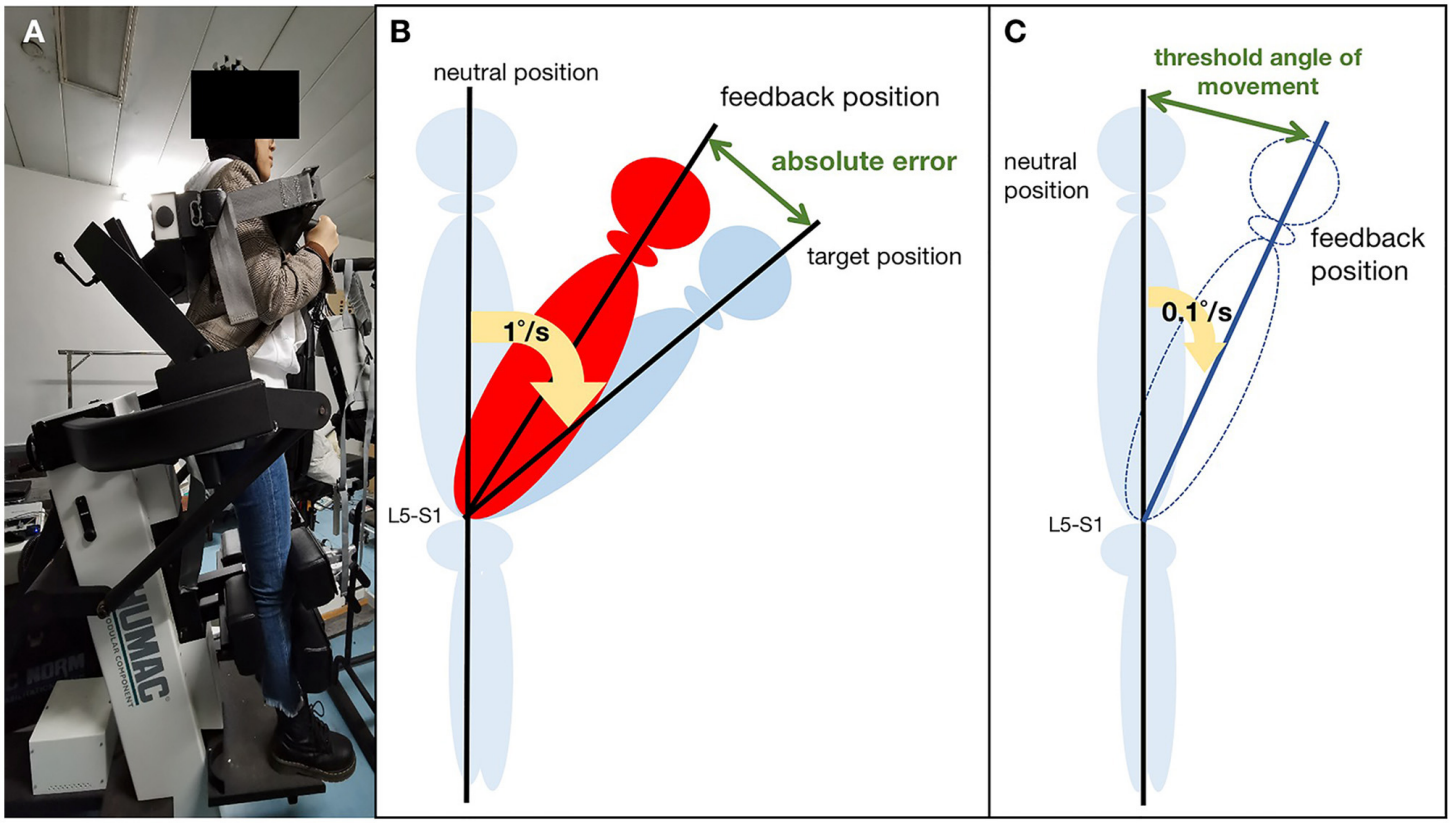
Lumbar proprioception measurement: **(A)** Schematic representation of the experimental setup; **(B)** Passive joint repositioning sense (JRS) test; **(C)** Threshold to detection of passive motion (TTDPM) test.

Passive joint repositioning sense (JRS) and threshold to detect passive motion (TTDPM) test were used to test lumbar proprioception. The JRS and TTDPM are commonly used to assess lumbar proprioceptive deficits with high intraclass correlation coefficients (18–20). Lumbar proprioception was assessed using the Humac Norm (CSMI, USA) with sagittal plane trunk attachment (Figure 1A). The Humac Norm was equipped with a software (HUMAC2013, USA), which allows passive velocities of between 1°/s and 0.1°/s, making it appropriate for assessing the JRS and TTDPM. Before testing, the participants were firmly secured to the attachment using straps in a standing posture to stabilize the sacral base, minimize hip and pelvic involvement, and ensure trunk movement consistent with the dynamometer. The participants wore blindfolds and headphones to limit their visual or auditory input.

##### 2.2.2.1. Joint repositioning sense test

The participants were first instructed to remember the target positions (15° and 35°). Then, they moved from the neutral position, passively guided by the motor at a slow, steady pace (1°/s). The motor stopped until they reached the target position and then maintained the ending position for 5 s. Subsequently, the participants passively returned to the neutral position. They practiced the procedure twice before the formal assessment. The procedure was repeated three times, and each participant requested feedback immediately after they reached the target position. Reposition error (RE) was measured in degrees, and only the absolute error (AE) was taken as a measurement (Figure 1B). We calculated the average of these assessments in the final analysis.

##### 2.2.2.2. Threshold to detect passive motion test

The Humac Norm passively rotated the upper body at a speed of 0.1°/s from the neutral position. The participants were asked to provide verbal feedback when they first perceived the trunk motion and pointed out the direction of motion correctly. The threshold angle of movement (angle of detection-starting angle) was recorded when the participant provided the correct direction (Figure 1C). Five assessments were performed, and the mean angle was used for statistical analysis.

#### 2.2.3. Proprioceptive postural control test

**Figure 2:**
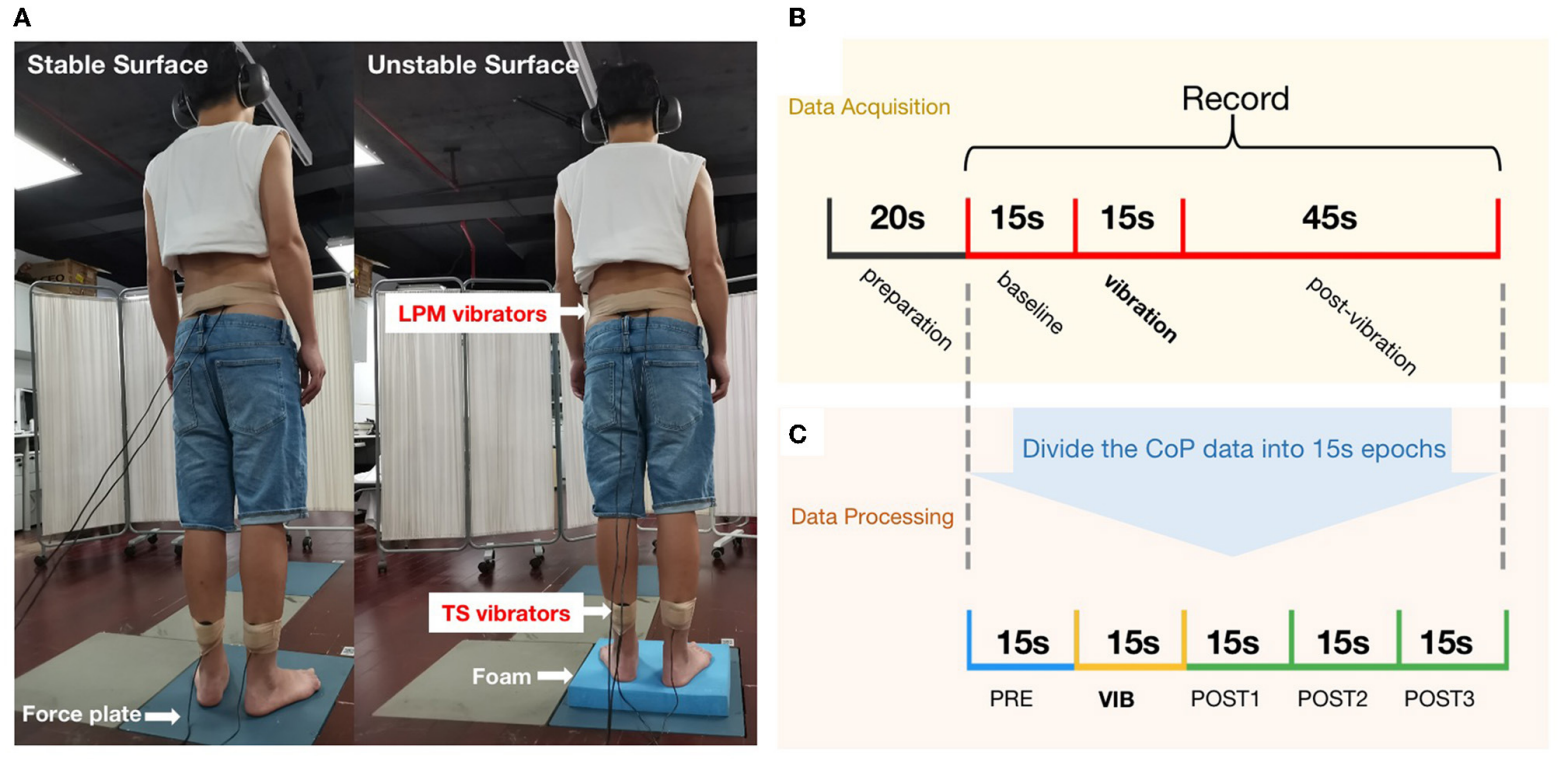
Proprioceptive postural control test: **(A)** Experimental setup: (left) standing on a stable supporting surface (force plate), and (right) standing on an unstable supporting surface (foam) with muscle vibrators on the triceps surae (TS) and the lumbar paravertebral muscles (LPM); **(B)** The experimental procedure for evaluating proprioceptive postural control; **(C)** The CoP data were analyzed using 15 s epochs and 15 s sliding windows.

To appraise postural stability and proprioceptive postural control, two supporting conditions were used: an upright standing condition on a stable support surface or an unstable support surface. Both conditions consisted of two muscle-vibration trials. Four custom-made muscle vibrators (frequency: 60 Hz) were placed bilaterally on the triceps surae (TS) and lumbar paravertebral muscles (LPM, at the L5-S1 level) (Figure 2A). The muscle spindle is particularly sensitive to vibration in 60 Hz (26). Therefore, four test conditions, including task 1 (stable support surface + LPM vibration), task 2 (stable support surface + TS vibration), task 3 (unstable support surface + LPM vibration), and task 4 (unstable support surface + TS vibration), were randomly performed. The participants rested for 3 min after each task.

Participants stood barefoot on a force plate (sampled at 1,000 Hz, AMTI, USA) and were asked to wear blindfolds and headphones to avoid the potential contribution of visual and auditory stimuli to balance. The participants were calmly in a standing position with arms to the side, and the center of the CoP was recorded after 20 s when it reached equilibrium.

Following the start of data collection, the participants stood for 75 s. Recordings began 15 s prior to vibration (“PRE”), the vibration was applied for 15 s (“VIB”), and the recording continued for another 45 s to assess post-vibration effects on balance (“POST”) (Figure 2B).

Data were analyzed offline using MATLAB (MathWorks Inc., USA). CoP data were filtered using a second-order low-pass bidirectional Butterworth filter. The cutoff frequency was set at 20 Hz, and bidirectional filtering increased the filter order to 4. CoP displacement is regarded as the most reliable parameter for evaluating postural balance control (21). The force platform signals of the CoP displacements along the anteroposterior (AP) and mediolateral (ML) directions were assessed separately.

Relative proprioceptive weighting (RPW) was used to evaluate proprioceptive postural control strategy (see Equation 1). An RPW value of 0 implies total reliance on LPM proprioceptive inputs. Conversely, higher values indicate increased reliance on TS proprioception (22). The AP or ML velocity was the average velocity of the CoP displacement in the coronal or sagittal plane. The sway path length (sway length) was the total length of the wandering CoP during each epoch. All measures were applied in a windowed (15-s epoch) manner to assess changes in CoP motion prior to, during, and after vibration. Data were analyzed using 15-s epochs. This includes the baseline epoch, the vibration epoch, and post 1–3 (post-vibration epochs), which started after recording the CoP and was shifted in time with 15 s intervals until 45 s after vibration to assess balance after vibration. This resulted in a set of five epochs (one baseline + one vibration + three post-vibration) for each participant, which was used for statistical analysis to assess group differences (Figure 2C).


$$\begin{array}{l} RPW\;TS/LPM=\left(absolute\;TS\right)/\left(absolute\;TS+absolute\;LPM\right) \end{array}$$

Equation 1 absolute TS: absolute value of mean sagittal CoP displacement during TS vibration; absolute LPM: absolute value of mean sagittal CoP displacement during LPM vibration.

### 2.3. Statistical analysis

Statistical analyses were performed using the SPSS software (Version 26, IBM Corp., Armonk, NY, USA). When Shapiro-Wilk tests showed that the data were not normally distributed, non-parametric tests were used for data analysis. Data are expressed as the median and interquartile range. Unless otherwise stated, parametric tests were used, and data were expressed as the mean and standard error. Nominal variables were compared using the chi-squared test. We performed a two-way repeated-measures analysis of variance (ANOVA) to test the differences in each of the CoP outcome measures of each task between groups. The groups and times were the factors. Sidak's multiple comparison tests were used to compare group differences among the five epochs. Statistical significance was set at *p* < 0.05 for all analyses.

## 3. Results

### 3.1. Participants

The demographic data of the 32 participants (*n* = 16 with CLBP, *n* = 16 without CLBP) are shown in Table 1. There were no significant differences in age, body mass index (BMI), and percentage of males between the two groups. Table 1 indicates that participants with CLBP had a moderate average pain intensity and mild disability.

**Table 1:** The basic characteristics of participants.

	**CLBP group (*n =* 16)**	**Control group (*n =* 16)**	***t* or χ2**	***p* value**
Age (year)	26.19 ± 4.63	25.56 ± 3.27	−0.441	0.662
BMI (kg/m^2^)	20.81 ± 2.18	20.94 ± 1.92	0.184	0.855
Gender (Male %)	18.8% (3)	50.0% (8)	3.463	0.063
Disability on RMDQ (0–24)	4.93 ± 2.59			
Pain identity on VAS (0–10)	4.16 ± 1.44			

CLBP, chronic low back pain; RMDQ, Roland-Morris Disability Questionnaire; VAS, visual analog scale.

### 3.2. Lumbar proprioception measurement

The non-parametric tests regarding JRS (flexion 15°, flexion 35°) and TTDPM showed that there were significant differences between the groups (Table 2). The reposition error and motion perception angles in patients with CLBP were higher than those in the healthy controls.

**Table 2:** Lumbar proprioception in people with and without CLBP.

	**CLBP group (*n =* 16)**	**Control group (*n =* 16)**	***z***	***p* value**
JRS on RE of 15° (degrees)	2.50 (2.50)	1.50 (1.42)	−2.635	0.008^**^
JRS on RE of 35° (degrees)	3.83 (3.75)	1.67 (2.00)	−2.916	0.004^**^
TTDPM (degrees)	1.97 (1.18)	0.68 (0.52)	−3.961	<0.001^***^

** *p* < 0.01,

*** *p* < 0.001, non-parametric test. CLBP, chronic low back pain; JRS, joint reposition test sense; RE, reposition error; TTDPM, threshold to detect passive motion test.

### 3.3. Proprioceptive postural control test

**Figure 3:**
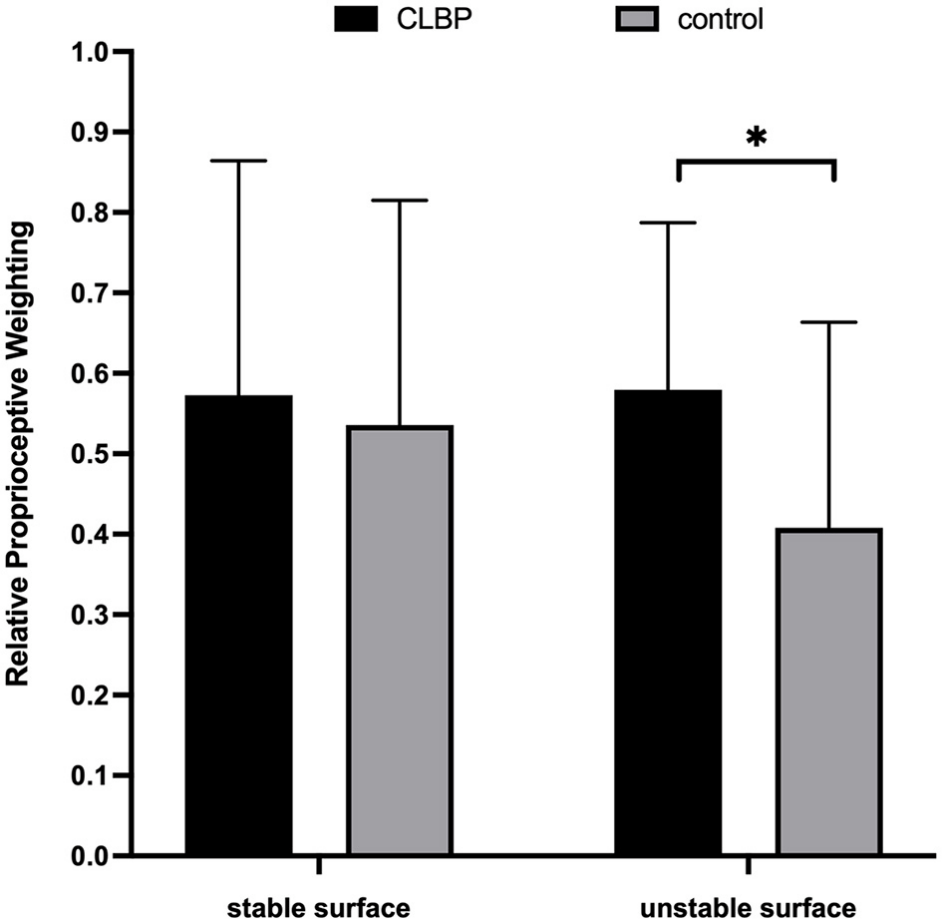
Column charts regarding the relative proprioceptive weighting scores of chronic low back pain (CLBP) and healthy control. ^*^p < 0.05.

The independent two-sample *t*-test indicated that CLBP (0.58 ± 0.21) demonstrated a significantly higher RPW value than healthy controls (0.41 ± 0.26) on the unstable support surface (*p* = 0.046) (Figure 3). Further, a non-significant difference was noted between the two groups on the stable surface (0.57 ± 0.29 vs. 0.53 ± 0.28, *p* = 0.715).

The results of the two-way repeated-measures ANOVA showed that all CoP parameters had no significant interaction effect under the four task conditions (Table 3). The main effects of time of sway length and average velocity in the AP directions were significant under the four task conditions. MLvelocity had significant time effects under task 1, 2, and 4. Sway length and AP velocity had significant group main effects under task 2 (average sway length: 206.73 vs. 154.29, mm; average AP velocity: 12.45 vs. 8.78, mm/s) and task 4 (average sway length: 341.23 vs. 265.59, mm; average AP velocity: 20.01 vs. 15.24, mm/s), and ML velocity had significant group main effect under task 4 (average ML velocity: 10.11 vs. 8.42, mm/s).

**Table 3:** Two-way repeated measures ANOVA for between group and time interactions of the center of pressure in patients with and without chronic low back pain.

**Task**	**Effect**		**Df**	**Sway length (mm)**			**AP velocity (mm/s)**			**ML velocity (mm/s)**		
				***F***	***p*** **value**	η^2^	***F***	***p*** **value**	η^2^	***F***	***p*** **value**	η^2^
1	Main	Group	1	2.192	0.149	0.068	2.177	0.150	0.068	0.714	0.405	0.023
		Time	4	8.023	<0.001^***^	0.211	7.503	<0.001^***^	0.200	5.646	0.001^**^	0.158
	Interaction		4	0.273	0.848	0.009	0.316	0.814	0.010	0.098	0.756	0.003
2	Main	Group	1	6.069	0.020^*^	0.168	8.688	0.006^**^	0.225	1.074	0.308	0.035
		Time	4	11.330	<0.001^***^	0.274	11.186	<0.001^***^	0.272	8.835	<0.001^***^	0.228
	Interaction		4	2.247	0.097	0.070	2.230	0.095	0.069	1.479	0.233	0.047
3	Main	Group	1	3.945	0.056	0.116	3.922	0.057	0.116	0.789	0.382	0.026
		Time	4	4.489	0.002^**^	0.130	4.899	0.002^**^	0.140	2.606	0.053	0.080
	Interaction		4	0.584	0.649	0.019	0.289	0.863	0.010	0.966	0.416	0.031
4	Main	Group	1	15.005	<0.001^***^	0.333	15.731	<0.001^***^	0.344	5.365	0.028^*^	0.152
		Time	4	23.714	<0.001^***^	0.441	17.611	<0.001^***^	0.370	24.286	<0.001^***^	0.447
	Interaction		4	2.536	0.057	0.078	1.911	0.128	0.060	2.350	0.077	0.073

* *p* < 0.05,

** *p* < 0.01,

*** *p* < 0.001. AP, anteroposterior; ML, mediolateral.

**Figure 4:**
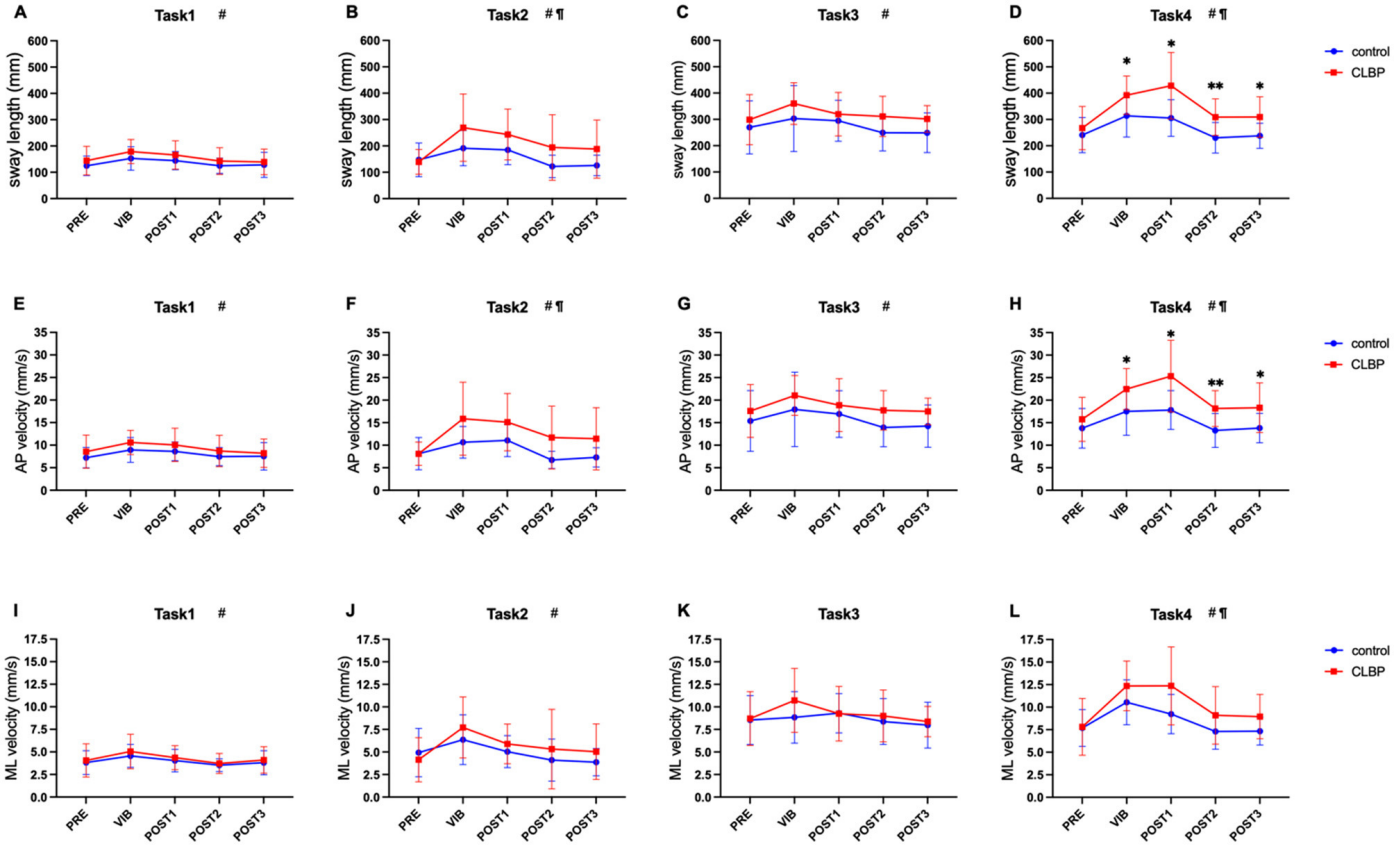
Two-way repeated measures ANOVA with Sidak's multiple comparisons test on each task (^*^vs. control), ^*^p < 0.05, ^**^p < 0.01. ^#^Significant main effect of time. ¶Significant group main effect. Horizontal axis: PRE (prior to vibration); VIB (during the vibration); POST1 (post-vibration 0–15 s); POST2 (post-vibration 15–30 s); POST3 (post-vibration 30–45 s). Longitudinal axis: **(A–D)** show the length of the sway path (sway length); **(E–H)** show the anteroposterior velocity (AP velocity); **(I–L)** show the mediolateral velocity (ML velocity). CLBP, chronic low back pain.

The results of Sidak's multiple comparison tests showed that there were significant differences in sway length and AP velocity during the vibration and post-vibration of task 4 (Figure 4). The sway length of the CLBP group was significantly longer than that of the healthy controls (VIB: 392.48 ± 72.82 vs. 313.86 ± 80.49, mm, *p* = 0.035; POST1: 428.42 ± 126.52 vs. 305.37 ± 69.46, mm, *p* = 0.012; POST2: 308.75 ± 69.29 vs. 230.24 ± 58.05, mm, *p* = 0.008; POST3: 309.47 ± 77.00 vs. 237.86 ± 47.63, mm, *p* = 0.020). Moreover, the CLBP group's AP velocity was significantly higher than the healthy control groups' (VIB: 22.48 ± 4.54 vs. 17.51 ± 5.29, mm/s, *p* = 0.039; POST1: 25.34 ± 7.96 vs. 17.81 ± 4.30, mm/s, *p* = 0.015; POST2: 18.15 ± 3.98 vs. 13.29 ± 3.77, mm/s, *p* = 0.007; POST3: 18.33 ± 5.52 vs. 13.80 ± 3.27, mm/s, *p* = 0.046).

## 4. Discussion

In this study, we investigated the changes in proprioceptive weighting and the effects of vibration of the TS and LPM on postural control in participants with CLBP. Our findings revealed that patients with CLBP had diminished lumbar proprioception and reweighting of proprioceptive input to the ankle. Compared to the control group, the CLBP group exhibited increased sway length and AP velocity during and after TS vibration. Notably, participants with CLBP showed higher sway of CoP during the first 15 s after the removal of the TS vibration on the unstable surface, which was not observed in the control group.

### 4.1. Joint position sense and kinesthesia

Our study found that the reposition error and the threshold angle of movement were significantly larger in the CLBP group. This indicated impaired joint position sense and kinesthesia in the lumbar region. The significant differences in proprioception agree with previous studies (4, 18). In particular, previous studies have reported that individuals with CLBP exhibited an increase in trunk reposition error during JRS experiments at various angles (15 degrees, 20 degrees, 30 degrees, etc.) (7). However, some researchers found no significant difference in reposition error between participants with and without CLBP (19, 23, 24). Notably, their method, in which participants underwent active JRS, differed from our passive JRS. Angela et al. performed JRS and found that the absolute error in active JRS was significantly smaller than that in passive JRS (19). During active JRS testing, muscles contract actively, and muscle spindles increase firing rates (25). The spindle is a specialized muscle fiber distributed in the skeletal muscle belly. It is activated when the length of the muscle fiber changes. The signals generated by spindles are the one of most important information for joint position sensing and kinesthesis (26). Proactive activation of spindles may compensate for proprioceptive deficits by providing additional sensory signals (27–29). This could result in no significant difference in active JRS between participants with and without CLBP. In addition, previous studies have found that adding vibration during JRS detection significantly increases the reposition error in healthy individuals. However, Brumagne et al.'s report confirms an opposite trend in individuals with CLBP, as they showed a smaller reposition error when vibration was added (30). The stochastic resonance-based vibration might enhance the reposition acuity, potentially contributing to the lack of significant difference in reposition error between CLBP and control groups. Further research is needed to fully understand the reasons for this observed phenomenon.

### 4.2. Proprioceptive reweighting

Our results suggested that the CLBP group demonstrated a significantly higher RPW value on an unstable surface. This indicates that patients with CLBP rely heavily on ankle proprioception and are unable to reduce their reliance even when signals from the TS become unreliable on an unstable surface. This phenomenon may be indicative of the deterioration in proprioceptive reweighting in patients with CLBP.

Through the process of proprioceptive reweighting, the central nervous system alters the weight of the ankle or lumbar proprioception to maintain postural balance. LBP may impair lumbar proprioception by inducing inflammatory responses (31, 32), disrupting proprioceptive signals from the trunk, and leading to decreased reliance on lower back proprioception for posture control (33). In addition, lumbar muscle atrophy could reduce spindle quantity, and the transformation of slow-twitch muscle fibers to fast-twitch fibers affects spindle function (34–36). LBP also causes the reorganization of the relative proprioceptive cortex. S1 (primary somatosensory cortex) mainly processes proprioception or a single sensory signal (37), while S2 processes dual-sensory or multisensory input (38). Studies have found that the lumbar representation area of S1 shifted inward in participants with CLBP (39), and the representation area of S2 (secondary somatosensory cortex) was blurred and unclear (40). The reorganization of these two regions affects proprioceptive perception and cortical processing. Moreover, Pijnenburg found that the white matter integrity of the superior cerebellar peduncle was reduced, which resulted in the neglect of position- and motion-related proprioception in the lower back (41). Altogether, the input from the lumbar spine decreases due to CLBP, and the central nervous system might increase the weighting of input from the ankle.

### 4.3. Proprioceptive postural control

We found that the addition of TS vibration resulted in greater CoP sway in participants with CLBP, particularly on an unstable surface. The muscle spindle, which is highly sensitive to mechanical stimuli, is responsible for the response to vibration (42). For postural control, afferent signals from the muscle spindle are crucial as they provide information about the position, movement, sense of force, effort, and heaviness (43). However, adding vibration may distort the proprioceptive information, creating a mismatch with other sources of afferent proprioceptive input and inducing unsteadiness in the balance control system (44). Low-amplitude vibration of the postural muscles could induce an illusion of muscle lengthening (45), which may alter the sense of upright and prompt posture adjustment (46). The extent of CoP displacement, which serves as an index of postural adaptation, reflects both the sensitivity of muscle spindles to vibration and the weightage that the central nervous system assigns to spindle input in perception and control of posture (13).

Excessive reliance on ankle proprioception may be the underlying cause of increased instability observed in CLBP patients during TS vibration. The transfer of proprioceptive signals to the ankle may result in a greater illusion of muscle lengthening in response to TS vibration, and this distorted information may cover up the proprioception information of muscle spindle provided by calf muscles to control balance. To avoid undesirable responses triggered by perturbation, the postural control system gates sensory inputs in accordance with the internal representation of the current posture (47). The increase of CoP parameters in CLBP patients during TS vibration suggests that this phenomenon may be related to the changes in the central nervous system's ability to process sensory information. It has been proposed that in the acute phase of LBP, this proprioceptive weighting change is an adaptation to decrease motor variability and reduce the input of injurious information to enable exploration of pain-free motor control solutions (48). However, if LBP persists and develops into a chronic condition, the proprioceptive weighting changes may result in central plasticity changes that can have a lasting impact on posture control. Prior studies have shown that the reorganization of S1 in CLBP may decrease the connection with M1 (primary motor cortex), which eventually impairs spinal postural control (49, 50). Additionally, the impaired connection between S2 and the premotor cortex affects sensorimotor integration (51, 52). A resting-state magnetic resonance imaging (MRI) study found that the functional connectivity between the S1, M1, supplementary motor area (SMA), and cerebellum decreased in patients with CLBP, which was correlated with poor sensorimotor performance (53).

Furthermore, we observed that removing the TS vibration, especially within the first 15 seconds after the end of vibration, led to greater balance disruption in participants with CLBP when standing on an unstable surface. This suggests that dynamic integration of sensory information could more challenging when there is an abrupt reduction in sensory input (removal of vibration) in balance tasks with greater sensory challenge, such as those with eye closure or unstable surfaces.

After the vibration is removed, a rapid reweighting of somatosensory information would be required to maintain balance (54). However, the proprioceptive information from the calf might contain more noise and less useful information than during vibration (55). The observation of greater CoP parameters for CLBP after removal of vibration suggest that CLBP may have difficulty effectively utilizing proprioceptive signals, particularly in the absence of visual feedback. Therefore, increasing sway could potentially generate more proprioceptive information to compensate for the loss of sensory input from other sources (56). Poorer balance control in CLBP after vibration removal may be attributed to an inferior ability to dynamically reweight the sources of somatosensory information and reduce the impact of vibration removal on balance. The duration of fluctuations may partly reflect recalibration of upright sense by dynamically reweighting sensory information (14). Our results show that even 45 seconds after the end of vibration, CLBP still exhibits greater instability than healthy individuals. It is necessary to delay the observation time in future studies to explore more postural control performance of individuals with CLBP.

Moreover, we observed that the between-group difference in sway velocity in the ML direction occurred only during task 4. This was due to the inconsistent somatosensory sensation in the direction of movement produced by different muscle contraction activities, with vibration TS and LPM mainly producing the illusion of movement in the AP direction (13, 57). Therefore, our results showed that the average AP velocity was greater than the ML velocity for the same task. Moreover, we considered the AP velocity to be a more sensitive and discriminative parameter in patients with CLBP.

### 4.4. Limitations

This study has certain limitations that need to be taken into account. Firstly, the sample size was relatively small, and the age range of participants was narrow, which may restrict the generalizability of the findings. Additionally, the majority of participants had a normal BMI, which may not allow for conclusive statements about the effect of obesity on lumbar proprioception in chronic low back pain patients. In this study, the average BMI of individuals with LBP was 20.94 ± 1.92 kg/m^2^, which falls within the normal range observed in previous studies on lumbar proprioception (18, 30, 58, 59). In some studies, LBP patients have been found to have higher BMI (60). Nonetheless, lumbar proprioception disorder was still observed in the CLBP population in this study, consistent with previous research (53–57). Future studies should perform subgroup analyses of CLBP patients with different body weights to better understand the impact of weight on CLBP proprioception. Moreover, larger and more diverse samples of participants should be recruited in future research to gain a better understanding of the relationships between body weight, age, and lumbar proprioception in chronic low back pain patients.

Secondly, the assessment of postural control was based solely on the CoP measurement using a force plate, which may not provide a comprehensive evaluation of postural control. Although CoP is a reliable tool for evaluating postural control, it only reflects the peripheral aspect of postural control and does not provide direct information on the central nervous system's contribution to postural control. Therefore, future studies could incorporate additional measures such as neuroimaging techniques to directly assess the central nervous system's involvement in postural control in chronic low back pain patients.

Thirdly, the assessment of lumbar proprioception was limited to the sagittal plane and did not include other positions, such as lateral bending and lumbar rotation, which may limit the comprehensiveness of the evaluation. In future studies, a wider range of positions should be tested to provide a more comprehensive assessment of lumbar proprioception. And in this study, we did not measure TTDPM and JRS during vibration conditions. In the future study, measuring TTDPM and JRS during vibration conditions could provide further insight into the effects of vibration on proprioception.

Finally, our study primarily focused on the contribution of the proprioceptive system to postural control, but it did not investigate the role of tactile sensation. It's worth noting that somatosensation comprises both proprioception and tactile sensation, which have been found to play independent roles in posture control and to even compensate for each other's weighting in healthy individuals (61). Furthermore, prior research has suggested that patients with CLBP may have impaired proprioception and reduced tactile sensation (48, 62), yet it remains unclear whether there's a sensory weighting shift between the two modalities in this population. Thus, future studies could incorporate measures of both proprioception and tactile sensation to offer a more comprehensive understanding of balance control in patients with CLBP.

## 5. Conclusion

In this study, we investigated the postural control performance in response to proprioceptive stimulation at the lumbar spine or ankle in the patients with CLBP. Our findings demonstrated that patients with CLBP exhibited impaired dynamic postural control in response to disturbances, which might be associated with the proprioceptive reweighting to the ankle. Consequently, further research investigating the mechanisms underlying postural control impairments in individuals with LBP should take into account the impact of alterations in proprioceptive weighting.

## Data availability statement

The raw data supporting the conclusions of this article will be made available by the authors, without undue reservation.

## Ethics statement

The studies involving human participants were reviewed and approved by the Human Subjects Ethics Subcommittee of the First Affiliated Hospital of Sun Yat-sen University (Grant No. 2021886). The patients/participants provided their written informed consent to participate in this study.

## Author contributions

XC wrote most of the manuscript and analyzed the data. JJY and ZMH wrote the rest and revised the main manuscript text. CHW directed the study and supervised the analysis. YL and RF measured the proprioceptive postural control presentations of the patients in the study. YZ and JJM examined the Lumbar Proprioception. WLL, QHY, and GFZ gave revised advice and helped with the language editing. All authors contributed to the study conception and design. All the authors have reviewed the final version of the manuscript, have approved it to be published, and have agreed to be accountable for all aspects of the work in ensuring that questions related to the accuracy or integrity of any part of the work are appropriately investigated and resolved.
